# Selective Argon Uptake and Structural Ordering in Carbon Honeycomb Nanostructures

**DOI:** 10.3390/ijms27146410

**Published:** 2026-07-19

**Authors:** Volodymyr Hamalii, Maksym Kabanenko, Dmytro Diachenko, Yuri Mastrikov, Eugene Kotomin, Nina Krainyukova

**Affiliations:** 1 B. Verkin Institute for Low Temperature Physics and Engineering of the National Academy of Sciences of Ukraine, 47 Nauky Ave., 61103 Kharkiv, Ukraine; gamalii@ilt.kharkov.ua (V.H.); kabanenko@ilt.kharkov.ua (M.K.); dyachenko@ilt.kharkov.ua (D.D.); 2 Institute of Solid State Physics, University of Latvia, Kengaraga Str. 8, LV-1063 Riga, Latvia; yuri.mastrikov@cfi.lu.lv (Y.M.); kotomin@latnet.lv (E.K.)

**Keywords:** carbon allotropes, carbon honeycomb, gas absorption, high-energy electron diffraction

## Abstract

This year marks the tenth anniversary of the experimental discovery and structural identification of carbon honeycomb (CH) structures. One of the most prominent properties of CHs is their unique high sorption capacity, which may play an important role in numerous applications, such as Li/Na batteries, hydrogen storage and carbon dioxide capture, as well as in the production of unique composites. In this paper we examine how atoms captured in CHs can be distributed inside CH channels using argon as a neutral probing material. Through a comprehensive structural analysis of the high-energy electron diffraction data, we demonstrate that argon atoms are not randomly positioned, but are rather arranged in specific clusters within the CH nanochannels. In the paper we propose two different original approaches to the structural analysis of pure and argon-filled CHs. We have also revealed that not all CH channels of various sizes are filled with argon, considering the spatial confinement in the thinnest channels to be one of the reasons for such a behavior. It was also found that external conditions may play an important role in filling CHs with gases, particularly at growing temperatures, when atoms gradually desorb, first from the channels of the largest sizes.

## 1. Introduction

**Figure 1:**
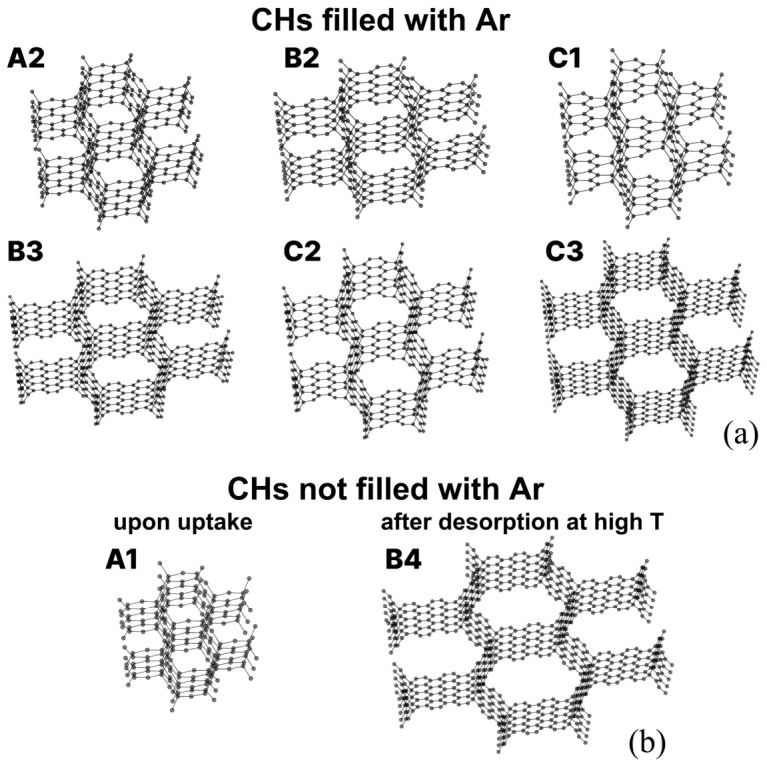
Three basic CH structures of types A, B, and C with different sizes designated by growing numbers and confirmed in the structural analysis of our experimental data to be filled with Ar (**a**). Two CH structures identified in the diffraction fitting procedure between experiment and modeling of empty CHs but were not found in comparison of experimental and calculated diffraction curves for CHs filled with Ar (**b**).

Carbon honeycombs (CHs) form a family of novel carbon allotropes that were experimentally discovered and identified by several structural methods ten years ago [1]. These structures were added to the group of relatively new carbon allotropes, which includes fullerenes [2], nanotubes [3], schwarzites [4,5], graphene [6], and some other forms that have been discovered within the last 40 years. For thousands of years, only two carbon forms, graphite and diamond, were widely known. CHs can be considered as three-dimensional graphene with a unique 3D cellular architecture resembling natural honeycombs. The walls of CHs are formed from monoatomic graphene nanoribbons that are aligned in one direction and joined by junction lines connecting three graphene planes at an angle close to 120° between two intersecting graphene nanoribbons (Figure 1). Inside the graphene walls carbon atoms in all CHs are intrinsically connected by *sp*^2^ chemical bonds as in graphene. However, along wall intersections atoms are arranged as in a diamond forming *sp*^3^ bonds inside tetrahedra centered on triple junction lines providing strong and stable framework configurations [7,8,9]. These configurations may exhibit different chiralities, channel sizes and tetrahedron orientations along junction lines where CH walls intersect. These give rise to three basic types A, B or C of CHs (see Figure 1), ensuring their high porosity with varied densities and sorption capacity already proved in several experimental studies [1,7,10,11].

Theoretical predictions suggest that CHs possess a variety of unique properties and useful applications. First of all, numerous theoretical studies and our own experimental observations have demonstrated the remarkable strength and stability of CHs [1,7,8,9,12,13,14,15,16,17,18,19,20,21,22,23,24,25]. Regarding their high sorption properties, which are their most prominent feature, their ability to capture and retain carbon dioxide has already been proven [11]. They are also considered promising candidates for hydrogen storage [26,27]. Furthermore, CHs exhibit low energy diffusion barriers for lithium or sodium along their channels as well as a high storage capacity, twice that of graphite for lithium storage. This makes them highly promising materials for Li/Na batteries [28,29,30]. After doping CHs are expected to exhibit excellent catalytic properties in hydrogen evolution reactions as well as in the oxygen and carbon dioxide reduction reactions [31,32]. CHs have a very high specific thermal conductivity, exceeding that of many metals and most semiconductors [20,24]. Their thermal conductivity exhibits a strong anisotropic effect, with values several times higher along the CH channels than perpendicular to the channel axes [33,34].

As has already been demonstrated, inert and molecular gases (such as Ar, Kr, Xe, CO_2_) can accumulate inside CH nanochannels due to physical absorption [1,10,11]. Neutral gas atoms in many cases are suitable model objects, and also they are well-known as ideal candidates for studying porous materials without destroying their structures. It is also worth mentioning that we have recently proved that metals such as aluminum can fill CH channels without significantly disturbing the matrix [35].

In this study we use argon as the anticipated proper filler in porous CH matrices in order to test not only their sorption capacities but also to analyze the structural peculiarities of both CHs and specific argon clusters formed inside CH channels in a confined geometry. We demonstrate that absorbed argon atoms, upon filling CH nanochannels, are not chaotically distributed, but form regular structures that can be identified using structural methods applicable to nanoclusters. The aim of the proposed study is to gain a basic understanding of the general sorption and structural properties of composites based on CHs and filled with various substances (using argon as a probing material). Such composites are expected to possess unique properties due to possible atomic ordering inside CH channels, which is important in particular for their potential applications.

## 2. Results

### 2.1. Experimental Observation of Ar Uptake in CHs and Desorption at Elevated Temperatures

The CH preparation procedure used in our studies is based on the mild vacuum sublimation of graphene patches of one atomic layer thick (though not single atoms) from graphitic rods that are thinned in their central parts and heated by an electric current. This process breaks the weak van der Waals bonds between graphitic monolayers during applied sublimation (avoiding overheating) while preserving the strong *sp*^2^ bonds within the graphitic planes, as described in more detail in previous papers [1,7,10]. The resulting graphene patches then fly and collide with each other, either in a vacuum or upon their deposition on prepared substrates. The colliding patches form triple connections with each other, with approximate angles ~120° between two neighboring patches along junction lines.

The detailed collision theory was developed earlier and suggested well-elaborated mechanisms for the formation of the main CH structural fragments [9]. These fragments are the basic constituents of CHs and include junction lines of three types, A, B and C, as shown in Figure 1 [7,9]. As demonstrated in [9], junction formations are highly energetically favorable, since many dangling chemical bonds around colliding patches are closed in such processes.

Formed fragments with triple connections between patches, together with single patches, either land as upright standing formations [36] on prepared flat surfaces covered by thin layers of polycrystalline NaCl or collide with fragments and patches that have already been deposited on prepared substrates. A small number of single atoms or pairs of atoms may fill empty positions. These processes result in strong and stable covalent configurations because open chemical bonds at the edges of the fragments and patches tend to close. Since the initial fragments and attached patches are upright standing, the CH nanochannels formed in numerous mutual collisions are strictly oriented perpendicular to the substrate. In these processes two basic chiralities in CH wall orientations with respect to junction lines and three types A, B and C of atomic connections in walls can be realized (Figure 1). Salt sublayers enable CH films (with a thickness of 120–140 Å) to be separated from substrates by dissolving NaCl in distilled water and captured on copper grids used in the high-energy electron diffraction experiments carried out in the wide temperature range 10–300 K. We applied the transmission high-energy electron diffraction (THEED) method to monitor and analyze in detail all structures and their transformations in our experimental studies.

**Figure 2:**
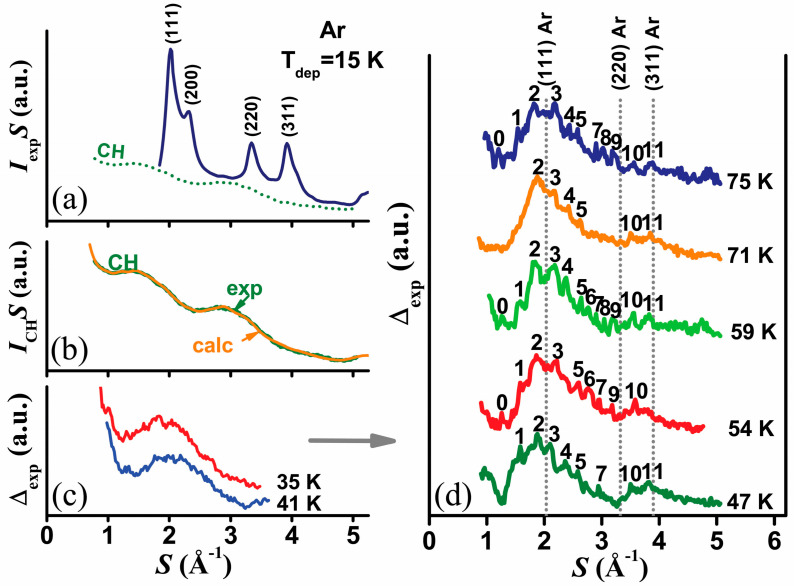
Experimental high-energy electron diffraction patterns from the as-deposited (at T = 15 K) argon film on the CH film (**a**) and from composites after filling CH matrices with Ar at growing temperatures (**c**,**d**). In the latter cases the contribution of the pure CH film (**a**,**b**) has been subtracted, and the curves are shifted along the vertical axis to make the details visible (**d**). The reliability factor *R*, derived from the fit between the experimental and calculated diffraction functions shown in (**b**), is 0.0032. The peaks of the bulk argon polycrystal diffraction are marked with Miller’s indices (**a**). Several reproducible minipeaks detected at different temperatures T in these experiments are labeled by numbers and discussed in detail in the text (**d**).

The methodology for filling CHs with different gases in situ inside the high-energy electron diffraction setup has also been developed and described previously [1,10,11]. In brief, gases are first deposited on CH films at cryogenic temperatures T as polycrystalline condensates with an average thickness of 150–200 Å. The diffraction pattern of polycrystalline argon deposited at 15 K is shown in Figure 2a along with the diffraction curve for the pure CH film. The latter is also shown separately in Figure 2b together with the calculated diffraction curve for CHs, as described in Section 2.3. The samples are then warmed up to temperatures a few degrees lower than the sublimation point T_subl_ of the deposited gas under study. Argon atoms diffusively penetrate inside CH nanochannels within a few minutes to form specific composites based on CH matrices filled with Ar. The full filling of all the available CH channels is ensured by a sufficient amount of deposited Ar condensates and temperatures high enough for active diffusion.

In the further analysis of obtained composites we consider the difference Δ_exp_ = *I*_CH+Ar_ − *kI*_CH_ between the diffraction curves for CHs after argon filling and for empty CHs, versus the modulus of the wave scattering vector S=4πsin(ϴ)/λ, where 2θ is the scattering angle and λ is the de Broglie wavelength of the electrons. The coefficient *k* < 1 and allows us to account for electron beam scattering and absorption by both CH matrices and argon atoms inside CH channels, while it is scattered only on CHs if we study pure matrices. The value of *k* is chosen so that always Δ_exp_ > 0 with a small additional positive term (not exceeding a few percent of the maximal Δ_exp_ value) for extrapolating the Δ_exp_ tails at the ends of the *S* intervals under consideration. To ensure uniformity, a similar coefficient *k* is also introduced for the applied model diffraction functions Δ_calc_, which are considered in Section 2.3.

Typically, argon sublimates at T_subl_ ~32 K from flat surfaces in an open geometry under vacuum conditions in our setup. In Figure 2c one can see a distinct residual signal in the form of a broad peak slightly above this temperature, which is associated with argon remaining in CH matrices. The broad peak is still visible at ~80 K, which is almost three times higher than the sublimation temperature of argon at flat open substrates. This fact may be explained by the stronger bonding of argon atoms inside the CH matrices compared to their interactions in its crystalline phase. Additionally, the high thermal conductivity of CHs [20,24] prevents the samples from overheating by the high-energy electron beam, ensuring gradual changes in sample states and reproducible diffraction patterns under fixed conditions. It should be noted that direct deposition of Ar atoms on CH films above T_subl_ was not observed, which is attributed to the high kinetic energy of Ar atoms in their gaseous phase, preventing argon capture and deposition on a substrate.

For the composites based on CH structures filled with Ar, the broad peak transforms and displays numerous smaller features resembling intensity oscillations when the temperature rises above 40 K, as shown in Figure 2d. At first glance, these signal oscillations exhibiting numerous minipeaks (shown and numbered in Figure 2d) look like some noises. However, when these curves are compared at different temperatures, it becomes apparent that the positions of these minipeaks coincide almost entirely. Some peaks are not visible at certain temperatures, but reappear in almost exactly the same positions when the temperature changes. Variations in the diffraction patterns may be caused by slight shifts in sample positions and consequently in the areas exposed to the thin electron beam when the temperature changes result in slight sample holder movements. Following an exhausting structural analysis of our samples and their diffraction patterns, we found that the most plausible explanation for the observed minipeaks is the structural ordering of Ar atoms inside CH channels of several types and sizes, as discussed in detail in Section 3. It should be noted that no similar minipeaks were detected in diffraction curves recorded for pure CH matrices, as shown in Figure 2b. This feature is also important and is discussed further.

### 2.2. Models and Diffraction Functions for Pure CHs and Filled with Argon Atoms

Structural analysis of nanosized objects typically involves comparing experimental and calculated diffractograms. The latter are produced using a number of model nanostructures built according to certain basic principles. Two sets of models were built for pure CH matrices and CHs filled with argon, and the relevant diffraction functions were calculated for further approbation in the analysis of the experimental diffraction data. A nearly complete set of pure honeycomb models of three types A, B and C, with a wide range of wall widths as well as both armchair and zigzag wall chiralities, was prepared previously [7] and utilized in the current analysis. These models were proven to accurately represent the structures obtained using our experimental production method, as detailed in Section 2.1 and in [7]. The pure CH models derived and selected in the current experimental study are analyzed in detail in Section 2.3, and are shown in Figure 1.

The other set of empirical models for argon filling the CH matrices was built by taking into account the physical sorption characteristics of all the relevant processes. Considering the interior geometry of the CH channels and all possible mutual atomic arrangements inside them, the distances between argon atoms were set to be close to, but not less than, the spacing between argon atoms in bulk argon crystals, equal to 3.75 Å. The minimal distances between argon and carbon atoms were set to 3.1 Å, though these distances could also be larger depending on the local available space in certain channels. The latter value is close to the Ar-C distance of argon atoms positioned above a graphene monolayer [37], although it is slightly smaller when considering the stronger interactions in fully filled CH nanochannels. Additionally, we prioritized Ar atomic positions with the maximal possible number of carbon nearest neighbors with respect to Ar atoms to maximize the bonding energies of Ar atoms with carbon walls upon adsorption. In all cases, we considered the maximum possible channel fillings, since the uptake and desorption processes presume full filling and complete emptying of the channels at the temperatures at which these processes occur. Indeed, upon uptake, a large amount of argon is deposited and the temperatures (slightly below the sublimation point) are high enough for active diffusion. When we reach the desorption point, specific to certain CH configurations, the Ar atoms removed first from the relevant channels make the remaining argon atoms less bonded. This allows the channels to empty quickly. Following these basic rules for Ar filling CH matrices, only a limited number of preferable atomic positions for Ar atoms inside CH structures can be obtained. This inevitably implies some kind of atomic ordering in periodic CH structures. In total, we built 20 models of argon filling various CH channels of different sizes and chiralities to be further used for a comparative analysis of calculated and experimental diffraction curves obtained at temperatures between 59 and 71 K. To enable comparison between experiments and modeling, relatively high temperatures, accessible in our measurements, were used to ensure sufficient structural relaxation of Ar clusters inside the CH channels.

**Figure 3:**
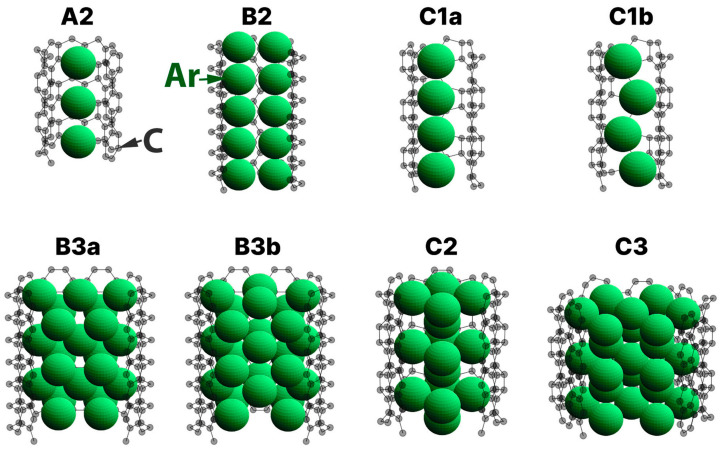
Structural models of CHs regularly filled with Ar in ordered atomic positions within CH matrices (as discussed in the text). The front parts of the CH walls have been removed to improve visibility.

In Figure 3 we show the models confirmed in our further analysis, described in Section 2.3, as reliable and existing in our composite samples based on CH matrices filled with Ar. In the thinnest channels of the A2 and C1 CH structures, we have found in the applied fitting procedure only monoatomic chains of Ar atoms interacting with all the surrounding CH walls and two nearest Ar atoms along the chains, which are parallel to channel axes. In the wider spaces between the walls of the CH structures B2, B3b and C2, the Ar atoms interact with the CH walls on only one side, but they also have more nearest argon atoms. In the widest channels of the C3 structure not all argon atoms interact directly with the walls, forming a row of Ar atoms in the channel center separated from the walls by argon layers. For the B3 structure, two possibilities for Ar filling comply with the basic rules formulated above and are considered separately (B3a and B3b in Figure 3). In the structure B3a the CH matrices are less densely filled with argon than in B3b; however, both structures were found in our samples.

Here we consider two basic methods that we used to calculate the diffraction functions inherent to certain CH model structures (both empty and filled with argon). These approaches differ significantly from the traditional methods used in the structural analysis of bulk crystals. The main approach was specifically developed to analyze nanostructures, i.e., any atomic formations ordered at the nanoscale. Although the calculated diffraction intensities differ for pure and filled matrices, both are based on the same initial approach. Specifically, we used the Debye formula [38] to calculate the diffraction functions:

(1) I=∑p,qNfpfqsin(RpqS)RpqS.

This is applicable to any structures with finite sizes. Here *R_pq_* is the distance between any two atoms *p* and *q* in a structure, with the atomic scattering factors for electrons *f_p_* and *f_q_* respectively, S=4πsin(ϴ)/λ, as described in the previous subsection. The summation runs over all pairs of atoms in any considered model structures.

First we consider the method of calculating diffraction functions applied to empty CH structures [7,10]. For monoatomic structures Equation (1) can be simplified:

(2) IcalcS=2Nf2∑n>msin(Srmn)Srmn.

**Figure 4:**
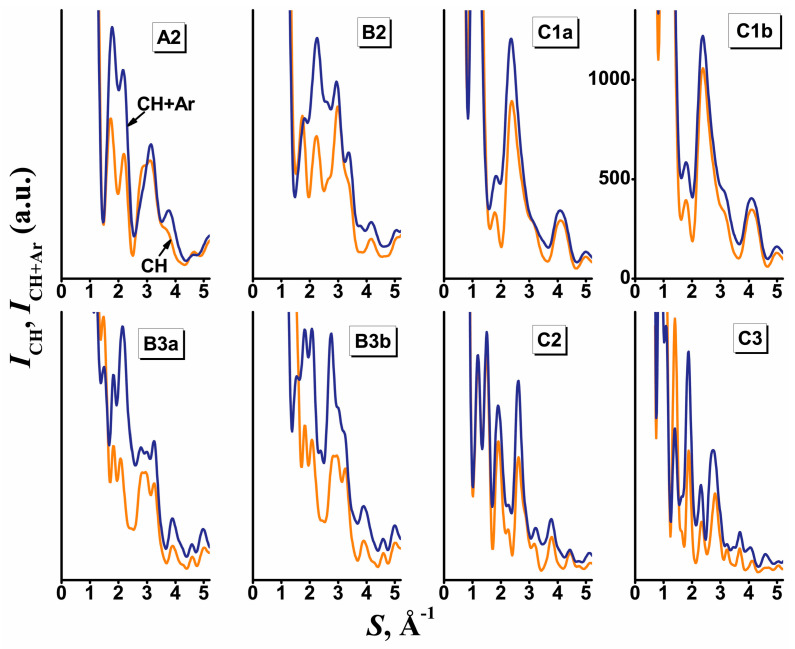
Calculated diffraction patterns for pure CH matrices of different types presented in Figure 1 (yellow) and for composites based on these CHs filled with Ar (blue) shown in Figure 3. These patterns were used in the fitting procedure with respect to experimental diffractograms.

This applies to any structural model comprising *N* atoms. The intensity here is normalized to one atom, and the factor of two together with the *n* > *m* condition is applied so that no pair of atoms is counted twice. Model diffractograms were calculated for a large set of CH structures [7] applying Equation (2) and were used for the pure CH analysis. Some particular diffraction functions of the structural models found in the analysis of the experimental data for pure CH matrices in this work are shown in Figure 4.

CH matrices filled with argon are two-component systems, and the diffraction functions of relevant models take into account this fact [11]. The general Formula (1) for the total intensity splits in this case into three terms:

(3) $$I_{A+B}=f_{A}^{2}I_{A}+f_{B}^{2}I_{B}+{f_{A}f_{B}I}_{AB}.$$

The first term describes one type of atoms, we mark as A carbon atoms, their amount is equal to *N*_A_. The second term refers to another type of atoms, which we mark as B argon atoms; their amount is *N*_B_. The third term describes pairs consisting of two different types of atoms A and B. *f*_A_ and *f*_B_ are atomic scattering factors for electrons of carbon and argon respectively. In Formula (3)

(4) IA=NA+2∑l>kNAsin(RklS)RklS,

(5) IB=NB+2∑n>mNBsin(RmnS)RmnS.

The first terms, *N*_A_ in Equation (4) and *N*_B_ in Equation (5), correspond to *l* = *k* or *n* = *m* respectively. Taking into account that the total number of atoms is equal to *N*_A_ + *N*_B_, we obtain

(6) IAB=2∑h>gNA+NBsin(RghS)RghS−2∑l>kNAsin(RklS)RklS−2∑n>mNBsin(RmnS)RmnS .

The factor of two before the sums together with the conditions such as *h > g* allows us again to take into account all pairs of atoms only once in the sums.

The calculated diffraction functions for composites based on CH matrices filled with argon in ordered positions (as shown in Figure 3) are presented in Figure 4 in a different color compared to pure CH matrices. We can see that after filling the CH channels with argon, the peaks on the calculated diffraction curves are mainly located in the same positions as for empty CHs but with essentially different intensities. This behavior implies that the CH matrices determine the basic symmetry of diffraction patterns for composites, while argon atoms, which occupy regular coherent positions within the CH matrices, as assumed during all model building, may only influence the redistributed intensities. For most curves the average high-energy electron diffraction intensities for composites are higher than those for pure matrices. This is natural, given the additional scattering associated with Ar atoms. However, it is remarkable to note that these excesses are not proportional to the relative Ar contributions, which may be considered a consequence and evidence for a certain ordering of Ar atoms in the CH channels. With a disordered Ar distribution inside CHs, one would only expect a monotonic contribution of high-energy electron scattering by argon into the diffraction intensities, which could only cause an intensity growth through the higher background.

### 2.3. Structural Fit Between Experiment and Modeling

We applied two different techniques for comparison of the experimental and calculated diffraction data for empty CH structures and composites built for CH matrices filled with argon, both of which are described further in detail.

Structural analysis of the diffraction data obtained for pure CH films was performed by comparing the experimental diffraction intensities *I*_exp_(*S*) obtained by the THEED method with the calculated intensities *I*_calc_(*S*) in their full form [7]

(7) IcalcS=exp−<u2>S2f2[11−t+∑kwkIcalc,k(S)].

As *I*_calc,*k*_ we apply the Debye formula [38] (shown earlier also as Equation (2), but now with the atomic scattering factor *f* for electrons shifted out of brackets in Equation (7))

(8) Icalc,kS=2Nk[∑n>msin(Srmn)Srmn]k

to be used in calculations of model diffractograms for a large set of nanosized CH fragments of three basic types A, B and C containing different numbers *N_k_* of carbon atoms in each model and applied in the further analysis (as discussed in Section 2.2). The diffraction intensities for all models were normalized to the number *N_k_* of atoms in every model. In Equation (8) *f* is the carbon atomic scattering factor for electrons and *r*_mn_ is the distance between any pair of atoms *m* and *n* in a structural model *k*. In this approach we varied also the mean-square atomic displacements <*u*^2^> and the probabilities *w*_k_ of the structural model *k* to be present in a sample to find the best fit between the calculated and experimental intensities *I*_exp_(*S*) assuming that $\sum_{k}w_{k}=1$ (see Figure 2b). The value *t* accounts for some part of atoms located at short distances from each other at the boundaries between different model fragments. These distances correspond to random connections of atoms, and therefore the oscillating terms corresponding to these distances (similar to those in Equation (8)) on average mutually compensate each other in $I_{calc}(S)$ (Equation (7)), contributing only to the monotonic term ~*f* ^2^. The fitting procedure between the experimental and calculated intensities (shown in Figure 2b) is based on the reliability factor *R* in the form

(9) R=∑SIexp−Icalc∑S(Iexp+Icalc),

where the summation runs over the *S* interval under study with the small step 0.02 Å^−1^ [7,10].

In the fitting we varied the contributions of different structural models to test their reliability in describing the experimental data. The fitting procedures between the experimental and calculated diffraction functions enabled us to select from the model sets the structures realized in our samples and their relative proportions.

In the diffraction analysis of CHs filled with argon atoms we initially used the calculated intensities of composites *I*_CH+Ar_ and empty CH matrices *I*_CH_, both shown in Figure 4. Then we consider the difference ∆_calc_ = *I*_CH+Ar_ − *kI*_CH_ between these two intensity dependences as a function of *S*, and refer to them further as reduced intensities. The coefficient *k* in ∆_calc_ (similar to ∆_exp_) accounts for the fact that for composites the total electron beam intensity is distributed between the CH matrices and argon atoms in CH channels upon scattering and absorption in a sample, while for pure CH matrices it is scattered only on carbon atoms in CHs. Typically, *k* < 1 and varies between 0.4 and 0.6 ensuring that always ∆_calc_ > 0 with a small additional positive term left (no more than a few percent of the maximal ∆_calc_ value) for possible extrapolation of ∆_calc_ tails at the ends of the considered *S* intervals. Note that the precise separation of scattering on CH matrices and Ar atoms absorbed in CH channels is impossible because these two processes are not additive due to the last term in Equation (3) describing possible coherence effects between carbon and argon atoms.

**Figure 5:**
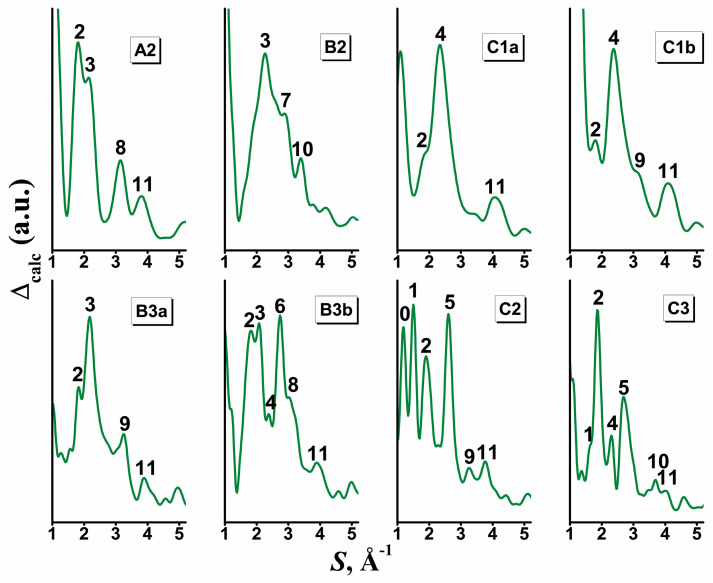
The difference in diffraction intensities ∆_calc_ between CH composites filled with Ar atoms and empty CH matrices (both shown separately in Figure 4) demonstrating many sharp peaks coinciding in their positions with experimental minipeaks marked by the specific numbers in Figure 2c.

The subtraction results are shown in Figure 5 for all cases of Ar filling confirmed in our analysis to be present in the prepared samples. For CHs filled with argon atoms, we consider the model diffractograms only the sets of peaks (such as shown in Figure 5), which are characteristic of every particular composite model structure. We take them as the two component sets *I*_n_(*S*_n_), where the n-th peak has an intensity *I*_n_ exactly in the peak maximum at *S* = *S*_n_. Such sets are characteristic and were built separately for all 20 model structures of composites based on CH matrices filled with argon. As it is discussed further in detail all experimental minipeaks (labeled by the same numbers as in Figure 2d) and calculated diffraction peaks were found to be very close in their positions, which are presented in Table 1. Note that all calculated diffraction minipeaks marked with numbers are presented in the experiment and vice versa.

**Figure 6:**
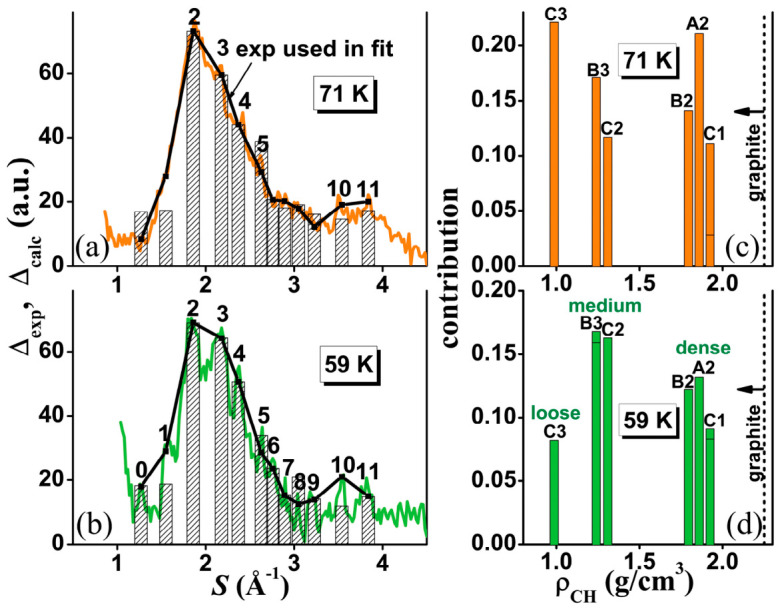
Experimental (exp, black) and calculated (column) diffraction profiles at 71 K (**a**) and 59 K (**b**) resulted from the fitting procedure with varied contributions of different CH composites filled with Ar atoms and shown in Figure 3. The reliability factors for these fits are *R* = 0.049 (59 K) and *R* = 0.057 (71 K). The full experimental curves Δ_exp_ are presented for comparison in colors: green at 59 K (**b**) and yellow at 71 K (**a**). Contributions of CH structures filled with Ar atoms found in the applied fitting procedure at 71 K (**c**) and 59 K (**d**) as functions of densities ρ_CH_ of the found contributing pure CH structures are also shown in Table 2.

The further fit was similar to the comparison between the experiment and the modeling considered for empty CH matrices. However, for composites only the calculated reduced intensities in the *S*_n_ positions corresponding to diffracted peaks obtained from the set of applied models were taken into account. Each model *k* contributed to the total distribution over the composite model structures with the probability *w_k_*, taking into account that $\sum_{k}w_{k}=1$. We minimize again the reliability factor *R* (shown as Equation (9)) by varying the probabilities *w_k_* but all summations are taken now over a fixed number of diffraction peaks for 20 initial composite models. The models found in this fitting procedure as contributed are shown in Figure 3. The fitting results for the calculated versus experimental reduced diffraction intensities and the relevant distribution functions over revealed structures (i.e., corresponding to non-zero *w_k_*) are presented in Figure 6 and Table 2. In Figure 6 the total calculated reduced diffraction functions are presented as columns with their positions corresponding to the diffraction maxima in Figure 5.

More specifically the comparison of experimental and model diffraction functions was made for the differences Δ_exp_ (between the experimental intensities for the CH matrices filled with Ar and empty CHs) shown in Figure 2d and calculated reduced intensities Δ_calc_ presented in Figure 5 for two temperatures 59 K and 71 K. As described above, in the fitting procedure we used the selective points *S*_n_ at the experimental curves corresponding to the maxima at the model diffraction functions, as shown in Figure 5.

For the precise analysis we chose the higher temperatures attained in our experiment to ensure sufficient atomic mobility, which may result in structural relaxation. The calculated diffraction patterns (presented by columns) strongly correlate with the experimental diffraction curves confirming the correctness of the assumptions applied to model building. In Table 2 we present the numerical contributions of all structures confirmed in our fitting procedure at 59 K and 71 K together with relative fractions of argon filling *N*_Ar_/*N*_C_ in all found structures relative to the number of carbon atoms *N*_C_ in CHs. We used a simple numerical procedure (based on the data presented in Table 2) to calculate the average filling fractions at these two temperatures, which were found to be 0.161 and 0.152 at 59 K and 71 K, respectively. Thus, this calculation demonstrates the gradual desorption of argon from CH channels at higher temperatures. Many other interesting features and correlations were found and discussed further in detail.

## 3. Discussion

One of the most interesting facts is our observation of numerous minipeaks noticed on experimental diffraction curves from CHs filled with Ar and well reproducible at different temperatures as it was described in Section 2.1. However, such minipeaks were not visible in the diffraction curves from empty CHs. Comparing experimental and calculated diffraction curves (Figure 5) and the data presented in Table 1, allowed us to find the most plausible origin of the minipeaks. We applied specific models of CHs filled with Ar atoms and found that the diffraction maxima in the calculated diffraction functions were positioned at the same *S* points (or very close) as the minipeaks observed in the experiment, as is demonstrated in Figure 5 with relevant minipeak numbers and in Table 1. This finding suggests that the minipeaks most likely originate from different well-ordered argon clusters formed inside the nanochannels of the CHs. These clusters have diffraction maxima that are specific to certain CH models filled with argon. This may be considered direct evidence that experimental diffraction patterns are superpositions of diffraction patterns from structurally different, well-ordered regions in CH films filled with argon.

While this conclusion seems natural, the question remains: Why do such minipeaks only appear in diffraction patterns for CHs filled with Ar, and why are they not visible in diffraction curves for pure CHs? This finding deserves to be considered and discussed more carefully. First, we can conclude that the reproducibility of the minipeaks in diffraction curves from CHs filled with Ar under varied conditions implies that Ar clusters are well-ordered inside CH nanochannels. A second reason is that argon clusters inside the CH channels are not only well-organized but also separated from each other by the CH walls. This separation prevents the ordered argon structures inside CH channels from being disturbed by other neighboring argon clusters if they are located at shorter distances between clusters. In empty CH structures, the boundaries between fragments of different A, B, and C types are not well-ordered, and CHs of different types and sizes must accommodate each other directly through poorly ordered intermediate regions. Diffraction from these regions makes it impossible to observe sharp peaks from the locally ordered fragments due to the contributions of many gradually transforming intermediate structures.

These specificities were considered in the different fitting procedures applied to pure carbon honeycombs and those filled with argon. For CHs filled with Ar atoms, only diffraction maxima on the calculated diffraction curves are sufficient to precisely describe all the experimental diffraction curves. These curves were found to be superpositions of diffraction from various channels with their specific Ar fillings. Furthermore, the experimental results specifically correlate with models featuring an ordered arrangement of Ar atoms within the CH channels. For empty CHs, we had to consider the entire experimental diffraction curves when comparing them to calculated diffractograms, not just for different model types, but also for numerous smaller structural fragments that may fill the space between CH domains.

Densities ρ of pure CHs gradually decrease (exhibiting the higher capacitance) with the growth of their channel sizes, particularly for the structures identified in our study, where they are 2.51 g/cm^3^ in A1, 1.92 g/cm^3^ in C1, 1.86 g/cm^3^ in A2, 1.79 g/cm^3^ in B2, 1.31 g/cm^3^ in C2, 1.24 g/cm^3^ in B3, 0.99 g/cm^3^ in C3 and 0.95 g/cm^3^ in B4. All values of density are smaller than the density of graphite (ρ = 2.26 g/cm^3^) except for the A1 structure, which is denser than graphite.

In distributions over specific composite structures shown in Figure 6c,d, one should notice that, despite differences in CH chiralities for A, B, and C types of CHs, all found CHs form three distinct groups depending on varying CH densities: dense, medium, and loose. These groups are distinctly separated from each other, mainly due to the discrete number of hexagons formed by carbon atoms along the widths of CH walls. This number is roughly similar for different types of CHs as their sizes grow.

Interestingly to note that at 59 K (Figure 6d and Table 2), both the B3a and B3b structures, which have different argon densities inside the CH channels, contribute almost equally to the resulting distribution. However, at 71 K (Figure 6c and Table 2), only the B3a structure with a lower argon density contributes, while the denser B3b structure is not detected. This may be due to the higher thermal expansion of argon clusters inside the CH matrices at T = 71 K compared to T = 59 K. Another obvious peculiarity of these distributions is that the argon atom content of the widest channel C3 increased by nearly a factor of three at T = 71 K compared to T = 59 K. This increase is likely due to the diffusive redistribution of atoms from the other channels, as well as the openness of this wide channel C3.

By comparing the sets of model structures derived from experiments with the help of applied analysis for empty CH matrices (Figure 1) and CHs filled with Ar (Figure 3), it is evident that all filled CHs are also present in the set of pure CHs as well. However, not all empty CH structures were found to be filled with Ar. This behavior may be attributed to two basic reasons: one is spatial confinement in the thinnest channels; the other is imposed by the external conditions, such as the sample temperature in CHs with wider channels. We revealed that the structure A1 shown in Figure 1 has a contribution to the set of models for pure CHs. However, the spatial confinement between CH walls in this structure makes it impossible to place Ar atoms there because they are too large to fit inside such thin channels. The other structure B4, which was revealed for pure CH matrices (see Figure 1), was also not found among the models filled with Ar in the analyzed temperature range 59–71 K. In this case the situation regarding CH sizes is opposite. The B4 structure has too large channels to keep Ar atoms bonded inside these channels; therefore, argon desorbs first from this structure at increasing temperatures compared to other CH structures with thinner channels. More specifically, this can be attributed to the weaker interaction between Ar atoms and CH walls when the atoms do not reside directly on the walls and are separated from graphene planes by other Ar layers. This situation is intermediate between denser CH matrices with stronger argon bonding with CH walls and flat graphene surfaces in vacuum, where Ar atoms sublimate at a well-defined T_subl_ under certain vacuum conditions. In general, we can formulate some conclusive rule that at elevated temperatures one can expect active argon desorption to occur first from the wider channels, where the bonding between argon and CH walls is weaker, and then at higher T from the thinner channels, where the interaction between argon and the walls is much stronger.

## 4. Materials and Methods

In this section, we summarize the materials and methods applied in our research.

To produce carbon honeycombs, we used pure graphitic rods that were heated in their central, thinned parts by an electric current to initiate the mild sublimation of graphene patches (but not single atoms), which preserve *sp*^2^ carbon bonding inside the graphene fragments. After the patches collide in a vacuum and are deposited on a substrate together with some single patches forming upright structures, we obtain CH structures as predicted by theory [9] and described in detail in Section 2.1.

The CH film was filled with argon in two steps. First, we deposited cooled gaseous argon onto prepared CH films at cryogenic temperatures. Then, we warmed the resulting condensates up to temperatures a few degrees lower than the typical sublimation temperature of argon for our vacuum conditions. The Ar atoms diffusively penetrated the CH structures, filling their nanoporous channels, as described in more detail in Section 2.1.

We used the THEED method on the high-energy electron diffraction setup produced at the electron microscope plant in Sumy, Ukraine. This setup was supplied by a cryostat manufactured at the B. Verkin Institute for Low Temperature Physics and Engineering of the National Academy of Sciences of Ukraine (NASU). This equipment operates within a wide temperature range of 10–300 K and was used for all structural studies and to monitor structural transformations in our experiments.

Our structural analysis was based on a comparison of experimental and calculated diffraction functions involving numerous structural models that we built according to transparent rules imposed by the physical adsorption character of filling CHs with Ar. We then conducted these studies applying analysis methods applicable specifically to nanostructures, which are described in detail in Section 2.

## 5. Conclusions

Carbon honeycomb (CH) structures are the first 3D graphene-based carbon forms revealed in experiment and structurally identified ten years ago [1]. These structures possess numerous unique properties, which have either been studied or predicted theoretically. One of the most prominent properties, both in scientific studies and in numerous practical applications, is their high sorption capacitance with respect to various materials. This has been proven for several gases [1,10,11], some metals [35], and is expected for Li/Na batteries [28,29,30]. It is also considered to be very promising for other practically important opportunities such as hydrogen storage [26,27] and capturing carbon dioxide from the atmosphere [11].

In this work we conducted the first detailed examination of the distribution of filling material atoms inside CH channels by using argon as a neutral probing material with a moderate atomic size. It is reasonable to expect that disorder or certain types of ordering could bring different properties in composites based on CH matrix hosts filled with various guest atoms.

Our study was performed using the high-energy electron diffraction method and compared to calculations. All of the calculated diffraction functions are based on structural models that were developed for three types of pure CH structures (A, B, and C) with various wall widths and two types of chirality, as described in the paper. These models are supported by the detailed theory of CH formation under the preparation conditions similar to those that we used [9]. Using the elaborated fitting procedures, we found almost perfect coincidence between the experimental and calculated diffraction patterns of both pure CH matrices and composites based on CHs filled with Ar.

The experimental diffraction patterns of CHs exhibit several specific features and overall differences between empty CHs and those filled with Ar. The most prominent feature is the observation of numerous minipeaks in the experimental diffraction curves of CHs filled with Ar that are not present in the diffraction functions of empty CHs. This striking difference can be attributed not only to the well-organized arrangement of argon atoms inside similar CH channels, but also to the separation of argon clusters formed inside the channels by CH walls. This separation prevents the ordered Ar structures inside CH channels from being disturbed by neighboring argon clusters that makes it possible to observe numerous reproducible minipeaks. These peaks can be considered superpositions of nearly independent diffraction patterns of Ar clusters formed within CH channels of various types and arranged similarly.

In empty CHs their multidomain structures are formed not only from well-defined structural fragments of three certain types A, B, and C, but also from the boundary regions where constituent fragments accommodate to each other through intermediate regions. Therefore, in empty multidomain CHs, all fragments of different types and sizes are not isolated, but rather they adapt to each other at the boundaries between individual parts, forming intermediate regions that exhibit gradual transformation from one structure to the other. These intermediate regions prevent the observation of the well-defined diffraction maxima, which are specific to every CH structure. This results in numerous mixed structural states.

In summary, we can conclude that saturating CHs with argon clarifies the entire picture of the Ar arrangement inside CH matrices and demonstrates the important role of Ar as a proper probing material for fully characterizing CH structures themselves.

Additionally, we have found that not all CH types are filled with Ar. For the densest A1 type of CHs Ar atoms are too large to fit inside their nanochannels. In addition, CHs with the wide channels, such as type B4, may not keep Ar atoms at high temperatures because argon atoms desorb owing to their weaker interaction with CH walls compared to CHs with thinner channels.

## Figures and Tables

**Table 1:** Experimental and calculated diffraction minipeak positions in *S* (Å^−1^) for different structures.

Peak Number	0	1	2	3	4	5	6	7	8	9	10	11
experiment	1.24	1.55	1.86	2.16	2.37	2.63	2.76	2.89	3.05	3.22	3.53	3.84
A2			1.83	2.14					3.07			3.82
B2				2.16				2.88			3.48	
C1a			1.84		2.33							3.92
C1b			1.79		2.39					3.18		3.91
B3a			1.83	2.18						3.24		3.90
B3b			1.82	2.12	2.39		2.74		3.04			3.90
C2	1.20	1.51	1.92			2.61				3.24		3.80
C3		1.61	1.87		2.33	2.65					3.58	3.91

**Table 2:** Contributions of CH structures filled with Ar atoms found in the applied fitting procedure at 59 K and 71 K, and fractions of argon filling *N*_Ar_/*N*_C_ in different structures with respect to the number of carbon atoms *N*_C_ in CHs.

Structures	C1a	C1b	A2	B2	C2	B3a	B3b	C3
59 K	0.083	0.091	0.132	0.122	0.163	0.168	0.159	0.082
71 K	0.028	0.111	0.211	0.141	0.117	0.171	0	0.221
*N*_Ar_/*N*_C_	0.083	0.083	0.063	0.100	0.179	0.218	0.250	0.250

## Data Availability

The datasets presented in this article are not readily available because the data are part of an ongoing theoretical study.

## Abbreviations

The following abbreviations are used in this manuscript:

CH(s)	Carbon Honeycomb(s)
THEED	Transmission high-energy electron diffraction

## References

[1] Krainyukova N.V., Zubarev E.N. (2016). Carbon Honeycomb High Capacity Storage for Gaseous and Liquid Species. Phys. Rev. Lett..

[2] Kroto H.W., Heath J.R., O’Brien S.C., Curl R.F., Smalley R.E. (1985). C60: Buckminsterfullerene. Nature.

[3] Iijima S. (1991). Helical microtubules of graphitic carbon. Nature.

[4] Lenosky T., Gonze X., Teter M., Elser V. (1992). Energetics of negatively curved graphitic carbon. Nature.

[5] Barborini E., Piseri P., Milani P. (2002). Negatively curved spongy carbon. Appl. Phys. Lett..

[6] Novoselov K.S., Geim A.K., Morozov S.V., Jiang D., Zhang Y., Dubonos S.V., Grigorieva I.V., Firsov A.A. (2004). Electric field effect in atomically thin carbon films. Science.

[7] Diachenko D.G., Krainyukova N.V. (2022). Structural variety and stability of carbon honeycomb cellular structures. Low Temp. Phys..

[8] Zhang Z., Kutana A., Yang Y., Krainyukova N.V., Penev E.S., Yakobson B.I. (2017). Nanomechanics of carbon honeycomb cellular structures. Carbon.

[9] Kawai T., Okada S., Miyamoto Y., Oshiyama A. (2005). Carbon three-dimensional architecture formed by intersectional collision of graphene patches. Phys. Rev. B.

[10] Krainyukova N.V. (2017). Capturing Gases in Carbon Honeycomb. J. Low Temp. Phys..

[11] Krainyukova N.V., Diachenko D.G., Kotomin E.A. (2024). Selective uptake and desorption of carbon dioxide in carbon honeycombs of different sizes. Low Temp. Phys..

[12] Karfunkel H.R., Dressler T. (1992). New hypothetical carbon allotropes of remarkable stability estimated by MNDO solid-state SCF computations. J. Am. Chem. Soc..

[13] Bucknum M.J., Hoffmann R. (1994). A Hypothetical Dense 3,4-Connected Carbon Net and Related B2C and CN2 Nets Built from 1,4-Cyclohexadienoid Units. J. Am. Chem. Soc..

[14] Umemoto K., Saito S., Berber S., Tománek D. (2001). Carbon foam: Spanning the phase space between graphite and diamond. Phys. Rev. B.

[15] Kuc A., Seifert G. (2006). Hexagon-preserving carbon foams: Properties of hypothetical carbon allotropes. Phys. Rev. B.

[16] Zhu Z., Tománek D. (2012). Formation and Stability of Cellular Carbon Foam Structures: An Ab Initio Study. Phys. Rev. Lett..

[17] Zhu Z., Fthenakis Z.G., Guan J., Tománek D. (2014). Topologically Protected Conduction State at Carbon Foam Surfaces: An Ab Initio Study. Phys. Rev. Lett..

[18] Gao Y., Chen Y., Zhong C., Zhang Z., Xiea Y., Zhang S. (2016). Electron and phonon properties and gas storage in carbon honeycombs. Nanoscale.

[19] Gua X., Pang Z., Wei Y., Yang R. (2017). On the influence of junction structures on the mechanical and thermal properties of carbon honeycombs. Carbon.

[20] Pang Z., Gu X., Wei Y., Yang R., Dresselhaus M.S. (2017). Bottom-up Design of Three-Dimensional Carbon-Honeycomb with Superb Specific Strength and High Thermal Conductivity. Nano Lett..

[21] Morris B., Becton M., Wang X. (2018). Mechanical abnormality in graphene-based lamellar superstructures. Carbon.

[22] Chen S.-Z., Zhou W.-X., Yu J.-F., Chen K.-Q. (2018). Nanoporous carbon foam structures with excellent electronic properties predicted by first-principles studies. Carbon.

[23] Wang H., Cao Q., Peng Q., Liu S. (2019). Atomistic Study of Mechanical Behaviors of Carbon Honeycombs. Nanomaterials.

[24] Wei Y., Yang R. (2019). Nanomechanics of graphene. Natl. Sci. Rev..

[25] Hu J., Zhou J., Zhang A., Yia L., Wang J. (2021). Temperature dependent mechanical properties of graphene based carbon honeycombs under tension and compression. Phys. Lett. A.

[26] Martínez-Mesa A., Zhechkov L., Yurchenko S.N., Heine T., Seifert G., Rubayo-Soneira J. (2012). Hydrogen Physisorption on Carbon Foams upon Inclusion of Many-Body and Quantum Delocalization Effects. J. Phys. Chem. C.

[27] Qin Q., Sun T., Wang H., Brault P., An H., Xie L., Peng Q. (2020). Adsorption and Diffusion of Hydrogen in Carbon Honeycomb. Nanomaterials.

[28] Liu J., Li X., Wang Q., Kawazoe Y., Jena P. (2018). A new 3D Dirac nodal-line semi-metallic graphene monolith for lithium ion battery anode materials. J. Mater. Chem. A.

[29] Shi L., Xu A., Zhao T. (2018). Three-Dimensional Carbon-Honeycomb as Nanoporous Lithium and Sodium Deposition Scaffold. J. Phys. Chem. C.

[30] Hua J., Zhang X. (2018). Theoretical prediction of honeycomb carbon as Li-ion batteries anode material. Eur. Phys. J. B.

[31] Lu Y., Ma Y., Zhang T., Yang Y., Wei L., Chen Y. (2018). Monolithic 3D Cross-Linked Polymeric Graphene Materials and the Likes: Preparation and Their Redox Catalytic Applications. J. Am. Chem. Soc..

[32] Jeon I.-Y., Choi H.-J., Choi M., Seo J.-M., Jung S.-M., Kim M.-J., Zhang S., Zhang L., Xia Z., Dai L. (2013). Facile, scalable synthesis of edge-halogenated graphene nanoplatelets as efficient metal-free electrocatalysts for oxygen reduction reaction. Sci. Rep..

[33] Wei Z., Yang F., Bi K., Yang J., Chen Y. (2017). Thermal transport properties of all-*sp*^2^ three-dimensional graphene: Anisotropy, size and pressure effects. Carbon.

[34] Chen X.-K., Liu J., Du D., Xie Z.-X., Chen K.-Q. (2018). Anisotropic thermal conductivity in carbon honeycomb. J. Phys. Condens. Matter.

[35] Kabanenko M.A., Hamalii V.O., Diachenko D.G., Mastrikov Y.A., Kotomin E.A., Krainyukova N.V. (2025). Pseudophysical sorption of aluminum in carbon honeycomb structures. Low Temp. Phys..

[36] Yuan Q., Hu H., Gao J., Ding F., Liu Z., Yakobson B.I. (2011). Upright Standing Graphene Formation on Substrates. J. Amer. Chem. Soc..

[37] Maiga S.M., Gatica S.M. (2018). Monolayer adsorption of noble gases on graphene. Chem. Phys..

[38] Warren B.E. (1969). X-Ray Diffraction.

